# Lightweight Pattern Matching Method for DNA Sequencing in Internet of Medical Things

**DOI:** 10.1155/2022/6980335

**Published:** 2022-09-08

**Authors:** J. A. M. Rexie, Kumudha Raimond, Mythily Murugaaboopathy, D. Brindha, Henock Mulugeta

**Affiliations:** ^1^Department of Computer Science and Engineering, Karunya Institute of Technology and Sciences, Coimbatore, India; ^2^Computer Engineering, Cybersecurity and AI School of Information Technology and Engineering (SiTE), Addis Ababa Institute of Technology, Addis Ababa, Ethiopia

## Abstract

An area of medical science, that is, gaining prominence, is DNA sequencing. Genetic mutations responsible for the disease have been detected using DNA sequencing. The research is focusing on pattern identification methodologies for dealing with DNA-sequencing problems relating to various applications. A few examples of such problems are alignment and assembly of short reads from next generation sequencing (NGS), comparing DNA sequences, and determining the frequency of a pattern in a sequence. The approximate matching of DNA sequences is also well suited for many applications equivalent to the exact matching of the sequence since the DNA sequences are often subject to mutation. Consequently, recognizing pattern similarity becomes necessary. Furthermore, it can also be used in virtually every application that calls for pattern matching, for example, spell-checking, spam filtering, and search engines. According to the traditional approach, finding a similar pattern in the case where the sequence length is *l*_*s*_ and the pattern length is *l*_*p*_ occurs in *O* (*l*_*s*_*∗l*_*p*_). This heavy processing is caused by comparing every character of the sequence repeatedly with the pattern. The research intended to reduce the time complexity of the pattern matching by introducing an approach named “optimized pattern similarity identification” (OPSI). This methodology constructs a table, entitled “shift beyond for avoiding redundant comparison” (SBARC), to bypass the characters in the texts that are already compared with the pattern. The table pertains to the information about the character distance to be skipped in the matching. OPSI discovers at most spots of similar patterns occur in the sequence (by ignoring *è* mismatches). The experiment resulted in the time complexity identified as *O* (*l*_*s*_. *è*). In comparison to the size of the pattern, the allowed number of mismatches will be much smaller. Aspects such as scalability, generalizability, and performance of the OPSI algorithm are discussed. In comparison with the hamming distance-based approximate pattern matching algorithm, the proposed algorithm is found to be 69% more efficient.

## 1. Introduction

Pattern matching is of two kinds based on the scenario for which it is applied: exact pattern matching (EPM) and approximate pattern matching (APM). An EPM is highly needed for the scenario in which the accuracy expected is 100%. For instance, when there is a search of a record in a database using a key value, exact matching is mandatory. Equally, APM finds its application in the fields like Bioinformatics, web search engines, text mining, intrusion detection system [1], and spam filtering. One of the interesting applications of string matching is text mining and which is discussed in [2] for extracting health-related information from Twitter messages.

In Bioinformatics, identifying similar patterns has a major part in sequence alignment, sequence assembly, a search of patterns in a DNA sequence, sequence comparison, and many more. For detecting a similar pattern, EPM has to be modified to ignore a few mismatches. The mismatch may be an insertion of a new character or deletion of a character or substitution by another character.

The problem of finding EPM was approached in different aspects and given solutions. The objective of the different tries on the same problem is to reduce the computation time taken for the solution. The time complexity for the worst case is in the order of the product of the length of the sequence and the pattern. Among the many solutions, the algorithm given by Knuth‒Morris‒Pratt (KMP) [3] proved to be having a linear time complexity, i.e., in the order of the length of the sequence alone. The proposed approach, optimized pattern similarity identification (OPSI), aims at finding the pattern similarity which needs to allow a few permissible counts of mismatches. The algorithm applies a similar computation of shift value as the KMP algorithm in the preprocessing stage. Then, in the proceeding stages, the algorithm identifies the pattern similarity with less than è mismatches.

In medicine, a term called next-generation sequencing (NGS) refers to a high throughput method of sequencing. The sequencing technologies have the fundamental characteristic of extensively parallelizing DNA molecule sequencing in flow cells [4]. After the alignment or assembly of short or long reads, DNA sequences can be utilized for any application. The short read is a part of the DNA sequence of length 100 to 300 base pairs produced by NGS technology [5]; and, long read lengths range from 500 bp to 800 bp [6]. NGS technologies have advanced to generate genomes that are longer than these lengths. The reads are mapped to the reference genome using bioinformatics analysis [7]. There are many DNA alignment algorithms proposed for merging the short reads into a single read and these algorithms are mainly divided into assembly and alignment of NGS data. Assembly of the short reads will be done in the absence of reference sequence [8, 9]; alignment will be done with the reference sequence as given in [10–13].

Read alignment is affected by some sequencing technology parameters, including the read length and error rate. It is essential to determine the actual location of each read, according to the reference. The exact location cannot be determined in advance, so the matching has to be done approximately [14]; the match must take into account a few mismatches and missing pieces within the reference sequence. The use of approximate matching would be unnecessary if there were no repeats in a genome and no errors introduced by a sequencing experiment. As long as there are sufficient read lengths relative to the genome size, exact matching can be applied to determine where the true locations are. Unfortunately, both assumptions are inaccurate: Eukaryotic genomes contain repeated elements, and each sequencing process has errors inherent in it [15]. The true location of a read is not accessible if it lies entirely within a perfectly identical repeat sequence. There are exceptions to this. Moreover, errors can occur in the reads, so the string matching needs to be approximated. There are two types of error models commonly used: 1. The hmamming distance manages the mismatches between sequence reads and the genomic region of interest, and 2. The edit distance takes into account indels and mismatches [16].

An alternate option to applying the edit distance, which handles all mismatches, insertions, and deletions equally, is to employ the weighted edit distance, which allows for the differentiation between mismatches and insertions, by assigning weights to errors. Even position-specific errors can be weighted differently when a weighted edit distance is developed by taking quality values into account. The quality value represents the probability that the base call will be mistaken [17]. Apart from these error models, affine gap can also be applied to longer indels, but at the expense of increased computational costs [18].

### 1.1. Motivation

There are similarities in genetic sequences among organisms (but different from one another). Because of point mutations, only a few positions are different between the organisms. These mutations can be nucleotide substitutions (another letter in the sequence) or insertions or deletions of some nucleotides. These sequence comparisons have been used to answer many biological questions, including the study of new Coronavirus strains, the prediction of genetic diseases, and the identification of cancerous cells. In order to do this, algorithms must be applied to align the sequences. For two sequences, this may not be a problem, but for hundreds or thousands, it may involve substantial computational effort.

Genome sequencing is a big reason for the current progress in genetics. Alternatively, it is known as a massively parallel sequencer. These technologies involve breaking the genetic sequence (that has a few billion nucleotides in an individual) into hundreds of nucleotides-long pieces and simultaneously sequencing them. Then, one has to reassemble the sequences, either by comparing the reads with a sequence for an organism, that is, similar (sequence mapping) or by assembling the reads based on similarity (sequence assembly). It is possible to achieve this through many sophisticated bioinformatic tools, including BWA [19], bowtie2 [20], TopHat [21], and Megahit [21], for example.

Blasting is the process of finding out what sequence belongs to a particular gene of a given organism by comparing the sequence to a large database. The BLAST [22] program is one of the many tools available to do this.

The crucial component of the algorithm in these entire DNA sequencing applications is pattern matching. Exact matches will not always yield the best results. Because of the source material and the process by which the sequences were created, there may be errors in the sequences. Even if the sequence and pattern are not identical, it will still be efficient to compare them, as long as the count of mismatched characters does not exceed a given threshold [23, 24]. The performance of the alignment algorithms is influenced by the performance of the pattern matching process. The impact on time motivated us to propose a time-optimized algorithm for approximate pattern matching.

The applications, which are depending on the similarity between the DNA sequences, greatly rely on the sequence alignment algorithms. Among these applications is the manufacture of drugs against Coronavirus. [25] Developed a method for aligning two Coronavirus sequences and identifying where mutations may have been occurring in the viral DNA. In the process of producing medicines to treat diseases, this information can be used. Furthermore, alignment algorithms are used for the correction of reading errors [26, 27]. KMP algorithm for pattern recognition, it helps to reduce the worst-case complexity, thus it helps to produce better alignment and better accuracy classification in the field of DNA sequence classifications [28]. To lower the make span rate, it is thought that the Internet of Health Things (IoHT) schedule must be balanced. For the optimum hybrid moth flame optimization (HMFO) job scheduling for cloud computing integrated with the IoHT environment over e-healthcare systems, we created a smart model technique in this study. Due to the anomalous changes in node energy levels, mobile nodes offer unreliable communication among themselves. It is so challenging to control such node actions that target node fails to gather packets [29–38].

The paper is organized as follows: Section 2 contains a literature survey; the EPM and OPSI algorithm are presented and demonstrated with examples in Section 3; Section 4 presents the result and discussion of OPSI algorithm, and Section 5 concludes the work presented.

## 2. Related Work

The focus of inventions of new pattern matching algorithms is to optimize the count of comparisons between the characters of the text and the pattern. Pattern matching can be mainly of two varieties, such as EPM and approximate pattern matching. The EPM problem is to find the occurrences of a pattern of length *l*_*p*_ in a text of length **l**_**s**_. Let the substring of length *l*_*p*_ in the text be called a window.

In the Brute Force approach [39], the pattern is aligned with the first window (i.e., the first *l*_*p*_ characters of text). Each character in the window is compared with the respective characters of the pattern. When a mismatch occurs, the window shifts by one position, i.e., the window starts at the second character and ends at the *l*_*p*_ + 1st character of the text. The character comparison continues between the window and the pattern. Since there is no preprocessing of the text or the pattern, this approach has the time complexity of *O* (*nm*) for the worst-case combination of text and pattern.

There are pattern matching algorithms using the hash-based method. One of the methods proposed earlier using hashing is the Rabin‒Karp algorithm [40]. The advantage of hash-based methods is that it encodes the characters into integers. Since the comparison has to be done only on the hash code and not on the strings, the comparison of numbers is time-efficient. However, the time complexity of the algorithm is *O* (*mn*); also, the computation of the hash code is complex in the Rabin‒Karp algorithm. To improvise efficiency, Zhao and Liu [41] suggested an algorithm that considers a partial number of bits in the binary representation of the character to compute the hash code.

Levenshtein algorithm is incorporated into the Rabin‒Karp algorithm and was proposed as revised Rabin‒Karp algorithm [42]. Hash distances are calculated for both patterns and text, resulting in an improved level of accuracy. Pattern and text are input parameters into the algorithm, and the Rabin‒Karp hash value is used as the output parameter. The Levenshtein method is applied to determine whether the sequence is similar. A similarity level is calculated by Levenshtein by comparing two input reads. And also, it is used to calculate the minimum effort needed to transform one input data to another string by doing minimum modifications. Using the combination of improved Rabin‒Karp algorithm, a Levenshtein algorithm, and additional check of quotient, it leads to improved accuracy and efficiency. However, the issue faced by hash-based algorithms is the collisions, i.e., more than one string is generating the same hash code.

Bloom filter supports a space-efficient data structure based on hashing. Bloom filters were used for representing the de Bruijn graph in less amount of space compared to the space originally required for storing the graph [43]. De Bruijn graph is applied for the assembly of DNA sequences produced by NGS. Najam et al. [44] proposed a pattern matching algorithm using multiple bloom filters. The method assures finding all positions of the pattern in the text in the compressed text itself.

To improve the performance of the Brute Force approach of pattern matching, improvised algorithms were proposed by preprocessing the pattern to take an optimized shift of the window. The KMP algorithm [3] and Boyer‒Moore (BM) algorithm [45] preprocessed the pattern to compute the shift position to be used to move the window when there is a character mismatch. Boyer‒Moore combines two approaches, bad character heuristic and good suffix heuristic. Each of these heuristics can be used independently of the other in order to find a pattern in a text. The pattern is processed and the two heuristics are mapped into different arrays. The pattern is shifted every time by the maximum suggested by each of the two heuristics. Each step consists of calculating the greatest offset suggested by the two heuristics. In contrast to other pattern matching algorithms, the Boyer‒Moore algorithm initiates the matching from the last character of the pattern.

Bad character heuristics are easy to understand. Assume that there is a character in a text that never occurs in a pattern. The pattern can be altered by changing the “bad character” to begin matching form substrings next to this character when it does not match (i.e., when a mismatch occurs at this character). As an alternative, it is possible that a bad character exists in the pattern; in this case, choose among bad characters in the text. As a result, the shift is likely to be higher than one.

Suffixes that have matched successfully are good suffixes. A mismatch that has a negative shift in bad character heuristics leads to an onward jump equal to the length of the suffix found in the substring of the pattern matched until the bad character. The average case complexity of both algorithms is *O* (**l**_**s**_). BM algorithm executes faster for an alphabet set of average size and lengthy patterns. But for the pattern of long length, preprocessing time is getting increased.

Reference [46] modified KMP algorithm to suit for searching an encoded pattern in Huffman encoded text. Since encoded contents are handled, there are chances for false matches in encoded text, i.e., the match found may not be an original match of the pattern in the text. He modified the KMP algorithm to be adaptable for handling binary strings. Also, since the BM algorithm is advisable for a larger alphabet set, KMP is chosen for the binary string.

**Figure 1:**
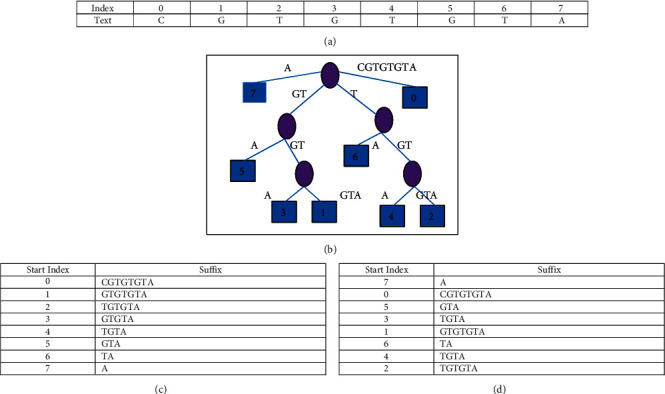
(a) Sample text and index. (b) Suffix tree. (c) All possible suffixes with start index. (d) Sorted suffixes.

As an alternate to calculate shift positions for the pattern, suffix trees were introduced and widely used in pattern matching algorithms [47–49]. The suffix tree is a data structure that will have the starting positions of each suffix of length one to the length of the text. An example is shown in Figure 1(b) depicting the suffix tree for the text, *t* = “CCACTGG.” The suffix tree has to be created for the whole text in which the pattern has to be searched. The amount of space needed for the suffix tree representation may be around 10 to 20 bytes per character [48]. Since the DNA sequence is very large in the range of billions, the space needed for the suffix tree will be more. Hence, there are many algorithms proposed for the compressed representation of suffix trees. Also, suffix trees were converted to suffix arrays to overcome the space issue faced by suffix trees [50–52]; but at the same time, efficiency was retained. The suffix array represents similar index information as the suffix tree but in the format of an array. The computation of the suffix array is exhibited in Figures 1(c) and 1(d).

The suffix array of the text (*t*), “CCACTGG,” i.e., SA (text) will be {7, 0, 5, 3, 1, 6, 4, 2}. Any pattern to be searched in the text will be a prefix of any of the suffixes if the pattern is present in the text. Hence, to search a pattern, the binary search is applied, and the comparison of the pattern begins with the suffix at the middle position of SA. For example, if the pattern (*p*) to be searched is “GTA,” *p* is compared with *t* [SA [3]], which is “GTGTA.” Since the comparison fails and *p* is lesser than “GTGTA,” the binary search will be continued in the first half of the sorted array. The complexity of the search is log **l**_**s**_ since the binary search is applied. However, since the suffix array has to be sorted, the complexity of the method is *O* (**l**_**s**_ log **l**_**s**_).

The research on pattern matching is not only into different data structures but also into suggesting hardware-related solutions to improvise performance. Özcan and Ünsal [53] proposed hardware-based solution for bitwise string matching and proved that it performs well.

The character-based algorithm, KMP, is having better time efficiency. The key idea of this efficiency is the computation of the shift value for the pattern. The same is applied for preprocessing the pattern and is utilized for identifying pattern similarity.

## 3. Research Methods

### 3.1. Exact Pattern Matching

Knuth et al. [3], (KMP), proposed an algorithm for EPM, which scans the characters of the given sequence only once from left to right, and the characters of the pattern were aligned to a character in the text according to the previous comparison. Hence, the time complexity will be in the linear order of the length of the sequence in which the pattern is searched for.

The algorithm makes sure that the characters of the sequence are scanned once. It is assured by preprocessing the pattern to calculate the shift position. The shift position indicates the next window for further comparison in case of any mismatch. The shift position is assigned such that the sequence of characters compared already with the pattern in the current window of the sequence will not be compared again if a mismatch occurs.

### 3.2. Preprocessing the Pattern

Let DNA sequence be of length *l*_*s*_ and the pattern of length *l*_*p*_. The straightforward algorithm for EPM makes a greater number of comparisons since there is an absence of observing the pattern before the comparison. Directly, the pattern is aligned at the left end of the sequence and character comparison starts. To enhance the solution, the pattern is scanned from left to right and observed for the repetition of any prefixes in the substrings. This observation is recorded in the shift beyond for avoiding redundant comparison (SBARC) table. The table consists of the shift value for the characters of the pattern. This assists in escaping from the redundant comparison in the occurrences of recurring characters.

**Figure 2:**
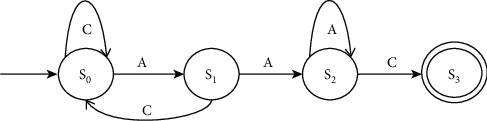
DFA for the pattern “AAC.”

Let DNA sequence be of length *l*_*s*_ and the pattern of length *l*_*p*_. The straightforward algorithm for EPM makes a greater number of comparisons since there is an absence of observing the pattern before the comparison. Hence, the time complexity of this solution is *O* (*l*_*s*_*∗l*_*p*_). For small *l*_*p*_, this is affordable. This becomes problematic as *l*_p_ increases. There is a lot of information available once it is checked whether *S* [*i*,…, *i* + *l*_*p*_ − 1] matches P or not, so we can determine whether *S* [*i* + 1,…, *i* + *l*_*p*_] fits *P*. There is one way to deal with this problem at a high level. This is to design a deterministic finite automaton (DFA) that depends on the pattern [54, 55]. The state diagram comprises *l*_*p*_ = |*P*| states. This automaton receives the sequence *S*. If a sequence of *j* characters from the pattern has already matched at the location, where they are currently in the sequence, the DFA guarantees that we will be in the *j*^th^ state. Now, the next two characters will be examined. The next state is reached if a match occurs, i.e., *j* + 1 characters are matched. An error can be resolved by going back to some earlier state. It is needed to make an intelligent decision about that earlier state. It will be an incorrect choice if the initial state in the DFA is selected. As based on the information about all possible states, the possible furthest back state has to be chosen. Suppose the pattern, *P* = AAC, then the DFA will be as shown in Figure 2.

**Figure 3:**
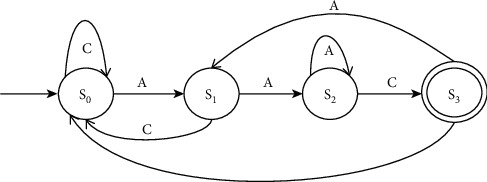
DFA for finding all repeated occurrences of the pattern “AAC.”

As soon as we see the pattern, the final state *(S*_3_) would be reached. As a result, the location at which the pattern *P* appears the first time in sequence *S*. When finding all instances of *P*, the DFA would need to be adjusted as shown in Figure 3.

The current location in *S* would be output every time the final state is reached, and then all instances of *P* in *S* could be output.

Having designed the DFA, it takes just *O* (*l*_*s*_) time to feed the sequence *S* and the positions in *S* into the DFA each time the final state is reached. The total run-time of the pattern matching algorithm would be *O* (*l*_*p*_) + *O* (*l*_*s*_) if building the DFA takes *O* (*l*_*p*_) time.

Let a pattern *P* be *p*_0_*p*_1_*p*_2_ … *p*_l*p − *1_, and *P*_*k*_ represents the prefix *p*_0_*p*_1_ … *p*_*k*_. In each prefix *P*_*k*_ there is one state *q*_*k*_. Being in the state *q*_*k*_ … the state of *q*_*k*_, the next state has to be decided when the character *c* is seen after *p*_*k*_. If *c* matches *p*_*k *+* *1_, then the state relevant to *P*_*k *+* * 1_ = *p*_0_*p*_1_ … *p*_*k*_*p*_*k *+* *1_ will be chosen. In any case, *c* is not the same as *p*_*k *+* *1_, a wise decision has to be made to choose the next state. It should not miss any possible match in the sequence.

Whenever a match begins in a region that already matches *P*, the match begins with any prefix of *P*; and, there has to be a matching pattern in *S* up to *k*. In order to avoid skipping a match in *S*, the longest such region is best. It is therefore necessary to identify the longest prefix of *P* that corresponds to *P*_*k*_. To be more specific, it will require dropping the fewest characters from Pk's beginning to get something that looks like a prefix of *P* again.

This observation is recorded in the shift beyond for avoiding redundant comparison (SBARC) table. The table consists of the shift value for the characters of the pattern. This assists in escaping from the redundant comparison in the occurrences of recurring characters. For computing such a skip, we focus on substrings of patterns that are prefix and suffix. A string must be different from the string itself in order to be a proper prefix. As an example, in the string “ACG,” the prefixes are “”, “*A*,” “AC,” and “ACG.” However, the proper prefixes are “”, “A,”and “AC.” In particular, we focus on substrings of patterns that are prefixes and suffixes. Whenever the prefix Pat [0…*i*] of pattern consists of *i* = 0 to *m* − 1, the shift value for the position *i* of the pattern, SVPat [*i*], stores the length of the maximum matching proper prefix, that is, also the suffix of the subpattern pat [0…*i*].

The algorithm for the computation of the SBARC table is given in Algorithm 1. The calculation is done as follows: the pattern is scanned from left to right; the shift value for the characters of the pattern is computed based on the repetition of any prefix of length 1 to *l*_*p*_ − 1 in Pat. If any such prefix is occurring for the next time, the shift value (SV) for the repetition will be related to the position of the same character in the prefix.

For example, considering the pattern “ACGACG,” the prefix “ACG” appears twice. Prefix “*A*,” of length 1, appears at position 4 again (at index 3); thus, index 3 has 1 for its shift value. Continuing the prefix “AC” ending at the fifth position (the index 4), the shift value for index 4 is 2; and prefix “ACG” of length 3 is appearing again till the sixth position (at the index 5), index 5 has 3 as the shift value. The shift value is assigned to each index position of the pattern and not to the characters in the pattern.

The shift value assigned for each position of the pattern is otherwise known as the longest proper prefix, that is, also a suffix. The important point to be observed here is that the prefix is the proper substring starting from the beginning of the pattern, but the suffix is considered for each substring ending at all possible positions of the pattern.

During the comparison of the pattern against the sequence, the search starts from the left end of the sequence. The pattern is aligned with the first *l*_*p*_ characters of the sequence. The character-by-character comparison is performed from left to right. When a mismatch is found after matching of few characters, a few characters of the next window are already known. Using this advantage, we do not have to compare those characters that are known to match anyway. Hence, when there is a mismatch, the shift value of the previous index of the mismatched position in the pattern is referred to. In the pattern, if any prefix is repeated as the suffix till the position of mismatch, the comparison of characters in the prefix need not be repeated with the sequence. Accordingly, shift value for each position of the pattern is assigned and the same is referred for continuing the comparison.

Comparison with the characters of the current window starts with Pat[j] with *j* = 0. Matching characters, Seq [*i*] and Pat [*j*], is continued and *i* and *j* are incremented, while Pat [*j*] matches Seq [*i*]. When a mismatch occurs, it is known that the characters Pat [0…*j* − 1] match with Seq [*i* − *j*…*i* − 1]. Additionally, it is true (from the compute_SBARC algorithm) that SV_Pat_ [*j* − 1] is the count of characters of Pat [0…*j* − 1] that are both proper prefixes and suffixes. As a result of these two points, it can be concluded that SV_Pat_ [*j* − 1] characters do not need to be matched with Seq [*i* − *j*…*i* − 1], because they will match anyway.

The core idea is not to go back to the previous characters in the sequence during a mismatch, i.e., the index used for the sequence will always be moving forward or idle, but not backward for sure. Only the index of the pattern will be getting increased or decreased based on the shift value. Hence, the EPM algorithm, presented in Algorithm 2, achieves a linear time complexity for pattern matching.

For the pattern matching process, the comparison of characters starts at index 0 of both Seq and Pat. The comparison continues towards the right linearly till the match of the characters is successful. Once a mismatch occurs between Pat [*j*] and Seq [*i*], the character of the pattern at the shift value of *j* − 1, i.e., SV_Pat_ [*j* − 1], is aligned to Seq [*i*] and the comparison continues.

### 3.3. Demonstration of the Algorithm with Example

**Figure 4:**
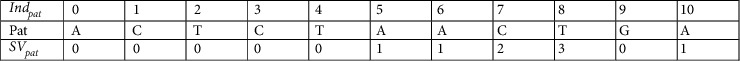
SBARC for the pattern.

Consider the example given in Figure 4 for the computation of the shift value of each position of the pattern. The shift value is assigned for each index of the pattern and not to the characters of the pattern. A pattern's index number is indicated by Ind_pat_, while the shift value is represented by SV_Pat_.

In the pattern “ACTCTAACTGA,” at index 5, 6 and 10 prefix A appear, hence, shift value 1 is assigned; prefix AC of length 2 is ending at index 7, hence, shift value 2 is assigned to index 7; prefix ACT of length 3 is ending at index 8, hence, 3 is assigned to 8; the method given in Algorithm 1 computes SBARC table of the pattern.

**Figure 5:**
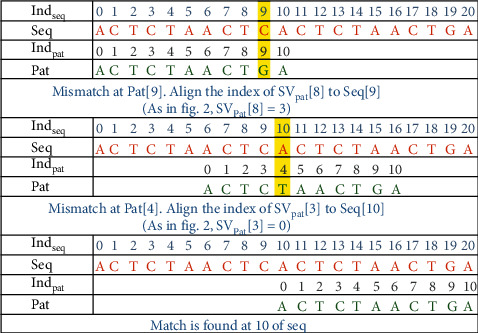
Demonstration of EPM.

The example is demonstrated in Figure 5 elaborates the process of pattern matching in a DNA sequence. Indseq and Indpat indicate the index positions of the characters in a sequence and in a pattern, respectively. The characters in the first window of 10 characters in the Seq are compared from left to right with the characters of the pattern. The characters from position 0 to 8 are matched successfully. Since Pat [9] is “*G*”and Seq [9] is “*C*,” a mismatch has occurred. As a result, it is apparent that the pattern is not present at position 0. A naive approach would shift the pattern into the window of index positions 1 to 11, and continue the comparison. The EPM algorithm uses the SBARC table to determine the index of the following window in order to optimize the comparison process. A new starting index is not explicitly assigned to the next window. Rather, it takes the index of the pattern character to be compared next to the 9th character from SVPat. Therefore, the character at the position of SVPat [8] in the pattern is aligned with Seq [9] and the matching process continues. Again, the Pat [4] is mismatched with Seq [10]. The next window is chosen by assigning the character at the position of SVPat [3] in the pattern aligned with Seq [10]. Followed by this, all the characters of the pattern are exactly matched from position 10 in the sequence.

In the sequence of length, the window of the pattern is shifted barely three times in order to identify the pattern. If the naive algorithm had been applied, the shifts would have been *l*_*s*_ − *l*_*p*_ + 1 times, which would yield 21 − 11 + 1 = 11. According to our analysis, the number of shifts has reduced drastically, reflecting the fall in the number of character comparisons in total.

### 3.4. Algorithm for Optimized Pattern Similarity Identification (OPSI)

While applying pattern matching in DNA sequences, there is a chance that the mutated pattern will be present in the sequence instead of the exact pattern. Hence, pattern similarity also needs to be enhanced to efficiently find out possible positions where the pattern is almost in the DNA sequence.

**Figure 6:**
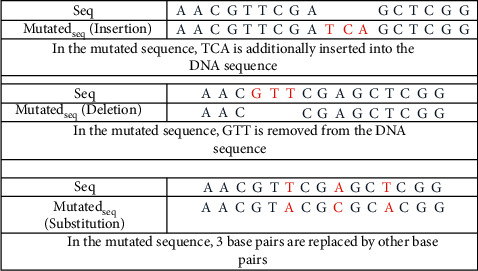
Example for mutation types.

APM can be made by allowing insertion, deletion, and substitution of characters for a given threshold value. The example in Figure 6 demonstrates the possible mutations in the DNA sequence.

OPSI algorithm identifies pattern similarity by allowing the mutation caused by substitution. During the search of a pattern in the DNA sequence, if the pattern is exactly matched with the current window of the sequence, the portion of the sequence matched with the pattern is not considered for approximate matching. Since the approximation is permitted, the time efficiency of the process depends on the number of mismatches considered. Hence, OPSI algorithm has a time complexity in the order of the product of the length of the text and the threshold for mismatches allowed (*O* (*l*_*s*_. *è*)). If the number of mismatches permitted is least considerable, then OPSI algorithm is having the time complexity in linear order. OPSI has shown an improvement as 69% in the execution time. With increasing thresholds for mismatches and sequence length, there will be an increase in time complexity.

**Figure 7:**
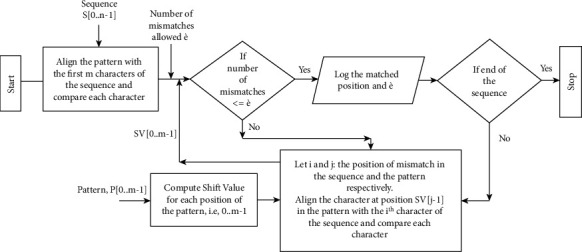
Flow diagram of OPSI.

When there is a mismatch between the characters of the sequence and the pattern, the pattern similarity is identified by allowing a specified number of mismatches. This threshold for mismatches is represented as *è*. The flow of OPSI method is illustrated in Figure 7.

**Figure 8:**
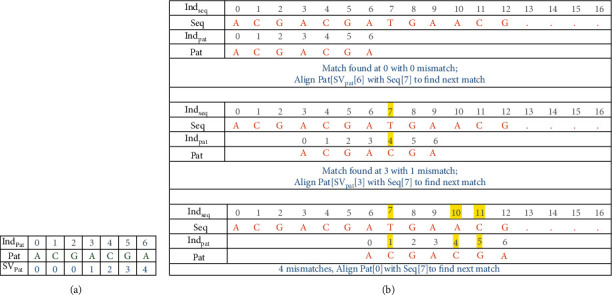
(a) SBARC table. (b) Finding pattern similarity.

The example in Figure 8 explains the pattern similarity permitting a maximum of three mismatches using OPSI. The EPM algorithm is followed the same way when there is a perfect match between the characters of the sequence and the pattern. When a mismatch occurs, the algorithm counts up the mismatch and proceeds as if there was no mismatch. When the count exceeds the specified acceptable number of mismatches, the algorithm alerts to switch to the shift value of the pattern, based on the index, where the first mismatch occurred in the current window of comparison. Hence, if the exact match is found, the algorithm proceeds, as the EPM algorithm works for the current window of characters. And during mismatch, the index of the text is also moved back to the position of the first mismatch.

OPSI algorithm, given in Algorithm 3, gets sequence (Seq), pattern (Pat), length of the sequence (*l*_*s*_), length of the pattern (*l*_*p*_), and the permitted number of mismatches (*è*) as input and returns the list of positions (Pos), wherever the pattern is found in the sequence with mismatches less than or *è* + 1. The comparison of characters starts as in the EPM algorithm. The matching process starts from the left end and moves to the right, character by character. When there is a mismatch and the count of mismatches till then is less than *è*, the count of error is updated and the comparison proceeds towards the right. If the index of the pattern becomes the length of the pattern, a pattern is found with a permitted number of mismatches and hence it is added to the output array, Pos.

If an exact match is found, the pattern index will update based on the last character of the pattern. If the pattern was found with mismatches or the number of mismatches overtakes *è*, then the index of the sequence is moved to the position, where the first mismatch occurred in the current alignment, and the pattern index is moved to the shift value of the character behind the position of the mismatch in the pattern.

## 4. Results and Analysis

### 4.1. Implementation and Experiment Design

OPSI was implemented in Python 3. Intel (R) Core (TM) i5-1035G1 CPU @ 1.00 GHz 1.19 GHz with 8 GB RAM. For the implemented work to be tested, data was taken from *Homo sapiens* chromosome Y, CM000686.2 in the NCBI library. Figure 8 shows a sample input sequence of 2000 base pairs (bp). In double-stranded nucleic acids, a base pair is formed by joining two nucleobases together with hydrogen bonds. Genes and organisms are described in terms of the number of base pairs contained in their genetic sequences as DNA is usually double-stranded. It is for this purpose that for every strand of DNA with a given sequence, there is an opposite strand containing complementary sequences as A complements *T*, and *G* complements *C*. According to Watson and Crick, the pair of researchers, who revealed DNA's structure, this complementarity was designated the Watson‒Crick pairing rule [56]. Based on this, the number of nucleotides in each strand equals the total number of base pairs.

**Figure 9:**
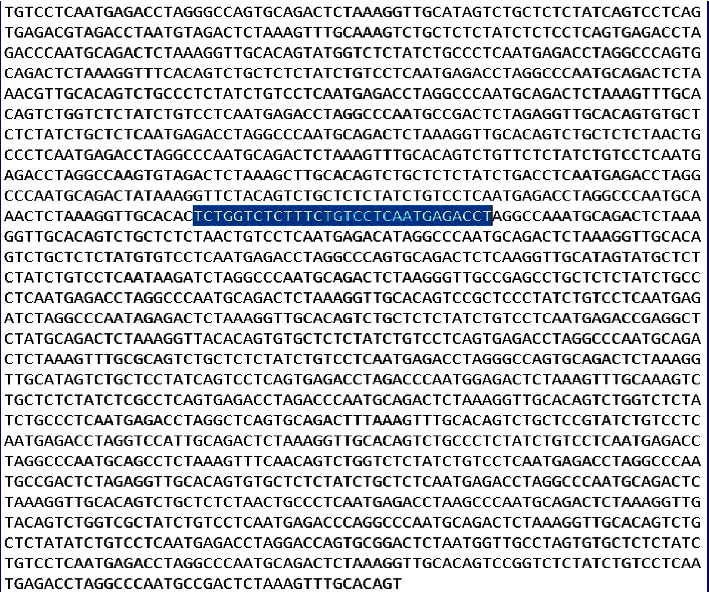
Sample sequence of length 2000 bp.

To experimentally demonstrate the output of EPM, the test input in Figure 9 was chosen as the sample sequence. The pattern “TCTGGTCTCTTTCTGTCCTCAATGAGACCT” of length 30 bp is given to be searched in the sequence. The pattern gets matched exactly starting from index 719 in the given sequence. The output obtained is given in Figure 8. The same input is then used for verifying the OPSI. The identification of exact pattern matching is done through the OPSI algorithm. If the same matching in EPM is identified by OPSI, it shows that the matching is not missed by the OPSI algorithm.

**Figure 10:**
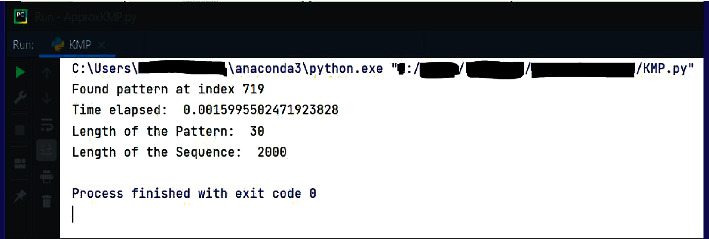
EPM search of “TCTGGTCTCTTTCTGTCCTCAATGA GACCT” in the sequence in Figure 7.

**Figure 11:**
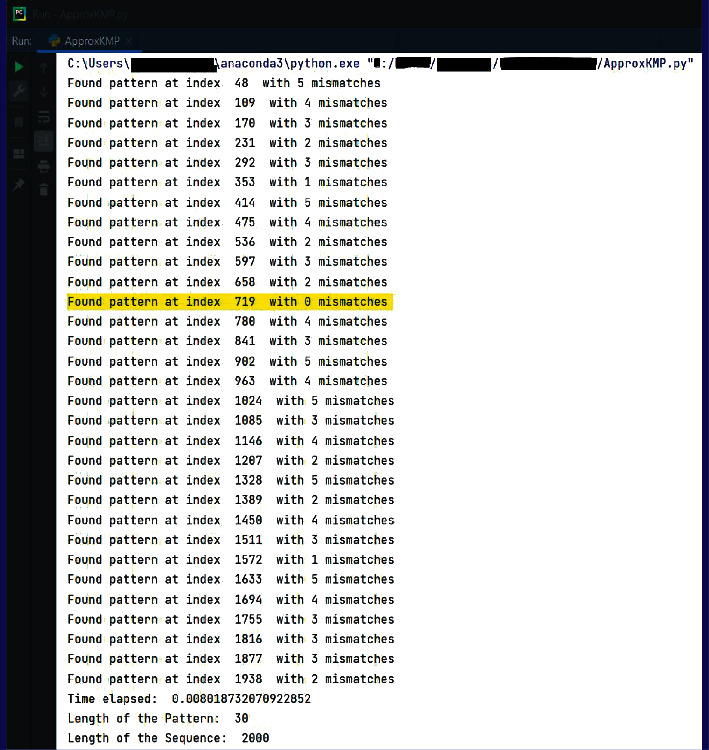
OPSI search of “TCTGGTCTCTTTCTGTCCTCAATGA GACCT” in the sequence in Figure 7.

OPSI algorithm is also executed for searching the same pattern “TCTGGTCTCTTTCTGTCCTCAATGA GACCT” of length 30 bp in the sequence in Figure 10, the tolerable number of mismatches is assigned as 5. The OPSI algorithm is applied to the same inputs as a check. If the same inputs are fed to the OPSI algorithm, it is expected that this exact matching position, as well as approximate matched positions, are to be located. All the approximate matching positions with 0 to 5 mismatches are expected to be identified. The output is shown in Figure 10. As a result, the exact matching position, and the starting indices of the pattern in the sequence, wherever it is approximately matched with a maximum of 5 mismatches, are listed out. The exact matching position 719 as given by the EPM algorithm is listed among the outputs received from OPSI algorithm and the same is highlighted in Figure 11.

### 4.2. Analysis of OPSI

In the compute_SBARC() algorithm, the pattern string is traversed at most once through the while loop. The variable *i* controls the number of iterations of the while loop. The variable *l* is used for tracking the length of the SV_Pat_ for the previous index. The variables *l* and SV_Pat_ [0] are initialized to 0. If Pat [l] matches Pat [*i*], *l* is incremented by 1 and SV_Pat_ [*i*] is assigned the incremented value. Whenever Pat [*i*] does not match to Pat [*l*] and *l* is not 0, *l* is updated to SV_Pat_ [*l *−* *1]. This process assures that the pattern is scanned once to its length. Hence the time complexity of the algorithm is *O* (*l*_*p*_).

There is only one while loop used in the EPM algorithm. The index variable, “*i*,”that controls the loop is initially set to 0, and it is incremented until “*i*” is equal to the length of the sequence. While this indicates that the while loop is executed ls times, it does not necessarily mean that it is executed exactly *l*_*s*_ times. This is because “*i*” is not incremented constantly throughout the while loop. There are few conditions in which “*i*” stays idle. In the event that the *i*^th^ character of sequence matches the *j*^th^ character of the pattern, “*i*” is incremented. If not, “*j*” is set to the SV_Pat_ of *j *–* *1 and the loop continues without an update to the index “*i*.” This is possible to a maximum of 2 *∗l*_*s*_ number of times. This means that the EPM algorithm has *O* (*l*_*s*_) time complexity.

Together, the compute_SBARC and EPM algorithm takes *O* (*l*_*s*_ + *l*_*p*_) time. Due to the shorter length of the pattern than the length of the sequence, the time complexity is bounded by *l*_*s*_. This leads to the conclusion that the finding exact matching has the time complexity *O* (*l*_*s*_).

OPSI algorithm behaves in a similar way when there are exact matches found in the sequence. When mismatches occur, the behavior of OPSI differs. The input scenario for the OPSI algorithm can be categorized into three cases.

**Case 1 .**

All exact matches of the pattern in the text: For every window alignment of pattern with the substring of text, if there are always exact matches found in the text, OPSI proceeds as same as the EPM algorithm. Hence, the time complexity will be linear for this case.

**Case 2 .**

Lesser number of mismatches than *è*: If the number of mismatched characters is less than the permitted count of mismatches, then, the next window of search is decided based on the shift value of the position where the first mismatch was found.

**Case 3 .**

Number of mismatches exceeding *è*: When the number of mismatches exceeds the count, then the search will be continued from the shift value of the first mismatched character in the present window of search.

Considering Cases 2 and 3, the position of the mismatch is resumed once the present window is processed. The shift value is used to select the next window for the matching process. Since the shift value assures that the compared pair of characters will not be compared again, the time complexity for the average case will be still linear. The worst case will occur when the number of mismatches in each comparison window exceeds *è*. In such a case, once the mismatch count exceeds *è*, further characters in the present window will not be compared; instead, the comparison window is immediately shifted. This leads to conclude that the time complexity of OPSI will be *O* (*l*_*s*_. *è*) in the worst case.

### 4.3. Experimental Results

The performance of the OPSI algorithm was experimented with and analyzed from three perspectives such as, (i) the preprocessing of the pattern is compared with the preprocessing of Boyer‒Moore for calculating the shift value, (ii) correlate with the EPM to prove the time complexity of OPSI, and (iii) comparison with the APM based on the hamming (edit) distance.

**Figure 12:**
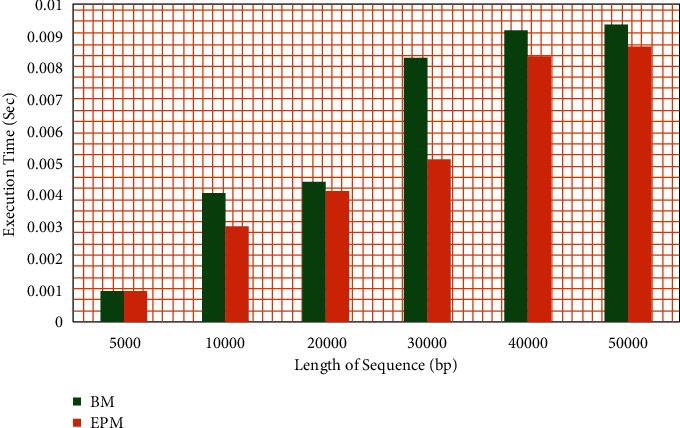
Execution time of preprocessing in Boyer‒Moore vs compute_SBARC.

According to the bad character heuristic, the Boyer‒Moore algorithm calculates the shift value during its preprocessing. Based on the presence of a prefix as a suffix, the compute_SBARC algorithm calculates the shift value. Both the algorithms were implemented in Python 3 and tested with patterns of size 5000 to 50000 base pairs. As shown in Figure 12, a graph is generated with data collected during the execution of both methods. Based on the graph, it can be concluded that the difference in execution time is negligible and that both algorithms perform in similar time frames.

**Figure 13:**
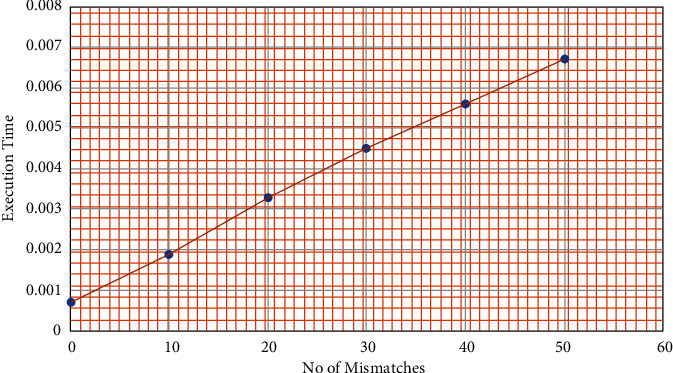
Execution time of OPSI vs number of mismatches.

OPSI algorithm was applied on a DNA sequence of length 6000 bp and a pattern of length 200 bp. The values for è are varied from 0 to 50 in an interval of 10. The algorithm for OPSI is applied with each of these *è* values repeatedly for five times and the average execution time is recorded.  Figure 13 shows that the execution time increases in the linear order of the growth of the number of mismatches.

**Figure 14:**
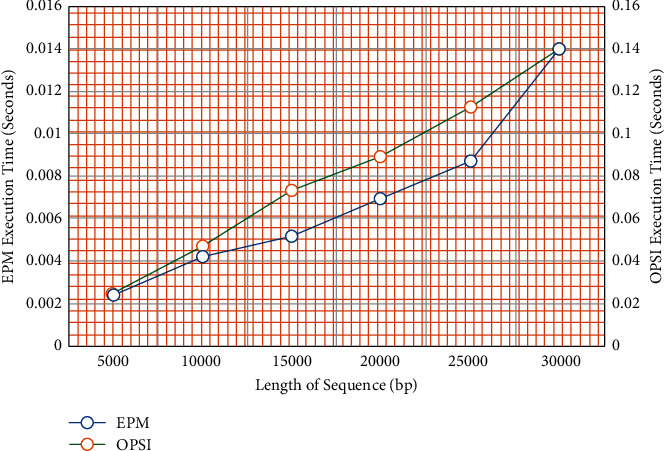
EPM vs OPSI.

OPSI and EPM algorithms are applied on DNA sequences of various lengths and the average execution times are recorded. The value of *è* is set to 10 and a pattern of length 200 is chosen for searching. The execution time is portrayed in Figure 14 for both the EPM algorithm and the OPSI algorithm. The execution time is taken as the average time of the multiple executions of the algorithms. It is observed that the execution time of Algorithm 3 is approximately 10 (=*è*) times the execution time of the EPM algorithm. Hence, supports the statement that the time complexity of OPSI is *O* (*l*_*s*_*∗è*).

**Figure 15:**
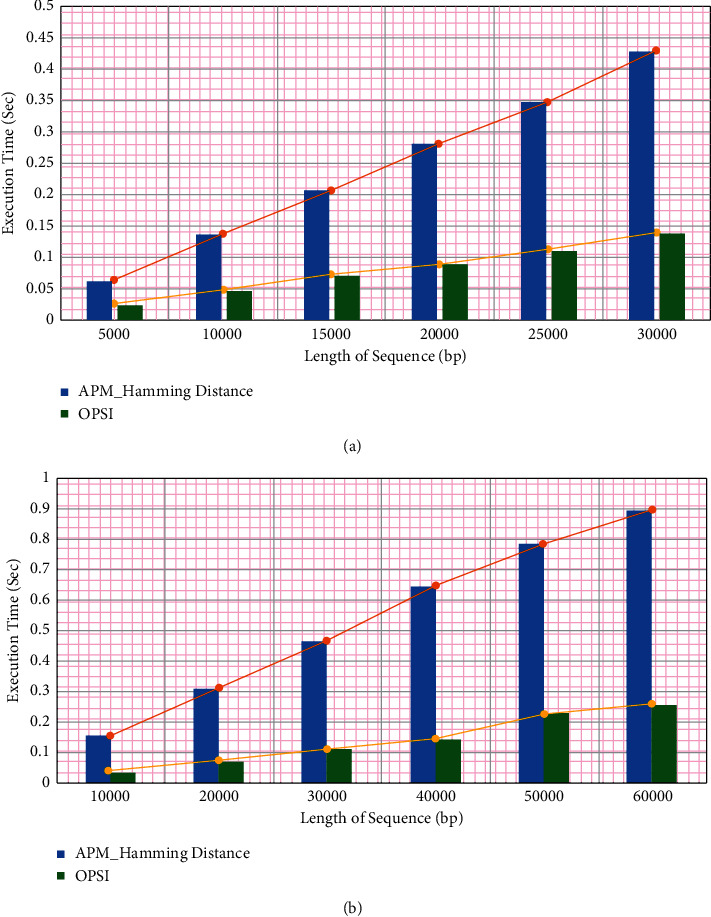
Hamming_Distance Based APM vs OPSI. (a) For DNA sequence. (b) For protein sequence.

The execution time of the OPSI algorithm is compared with the APM algorithm based on hamming distance [23]. The OPSI algorithm is not limited to DNA sequences, as it can be used with any alphabet. This has been demonstrated by applying it to protein sequences as well. The data for DNA and Protein sequences are taken from https://pizzachili.dcc.uchile.cl. A pattern of 200 characters is searched using the algorithms. The average execution time is observed for different lengths of the sequences for both algorithms. The graphs are given in Figures 15(a) and 15(b) report the time taken by OPSI and hamming distance-based APM for searching the pattern in increasing lengths of sequences. According to the difference between the execution times of OPSI and the other algorithm, OPSI algorithm is more efficient.

The improvement in the performance of OPSI is analyzed by comparing the slopes of the lines shown in Figure 15. Considering that the lines are not straight, slopes between every two points are measured. The average of these slopes is calculated and applied for computing the percentage of improvement in the performance of OPSI for APM. It is found to be 68.96% in the case of DNA data set and 70% for Protein sequence. On average, the execution time was improved by 69.48%.

## 5. Conclusion

Pattern matching is an important and frequent function in any field where a large amount of data is handled. Many algorithms are being proposed by researchers for improving the performance of the search. OPSI finds the positions of the pattern in the text by allowing a specified number of mismatches.

Since the approximation is permitted, the time efficiency of the process depends on the number of mismatches considered. Hence, the OPSI algorithm has a time complexity in the order of the product of the length of the text and the threshold for mismatches allowed (*O* (*l*_*s*_. *è*)). If the number of mismatches permitted is least considerable, then the OPSI algorithm is having the time complexity in linear order.

As compared to the APM based on hamming distance, OPSI has shown an improvement of 69% in the execution time. OPSI possesses scalability because there are no limits on the size of the inputs such as the threshold for mismatches, sequence length, and pattern length. With increasing thresholds for mismatches and sequence length, there will be an increase in time complexity.

In addition, because OPSI is generalizable, it can be applied not just to DNA sequences but also to any string-matching problems. This algorithm calculates the shift position based on the prefix. In future work, this work can be improved by integrating suffix-based methods such as the BM algorithm.

## Figures and Tables

**Algorithm 1:**
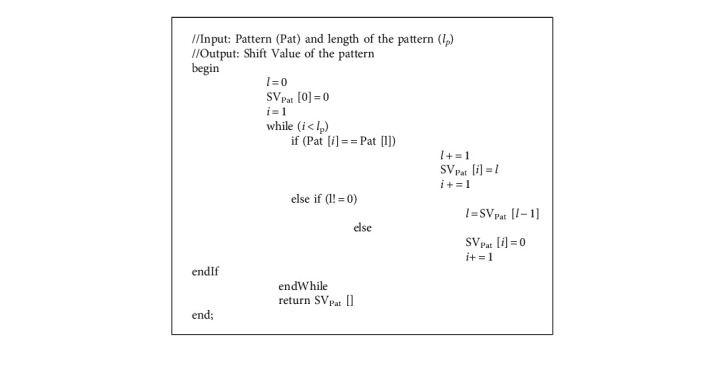
compute_SBARC(Pat, *l*_*p*_).

**Algorithm 2:**
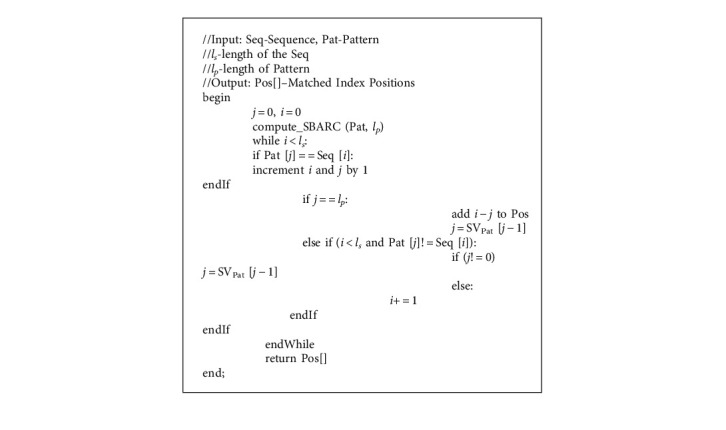
EPM (Seq, Pat, *l*_*s*_, *l*_*p*_).

**Algorithm 3:**
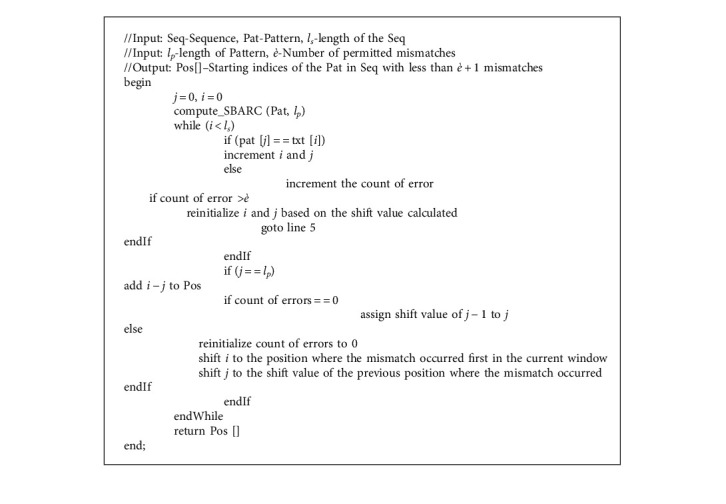
OPSI (Seq, Pat, *l*_*s*_, *l*_*p*_, *è*).

## Data Availability

The datasets used and/or analyzed during the current study are available from the corresponding author on reasonable request.
